# SIRT4 and Its Roles in Energy and Redox Metabolism in Health, Disease and During Exercise

**DOI:** 10.3389/fphys.2019.01006

**Published:** 2019-08-09

**Authors:** Yumei Han, Shi Zhou, Sonja Coetzee, Anping Chen

**Affiliations:** ^1^School of Physical Education, Shanxi University, Taiyuan, China; ^2^School of Health and Human Sciences, Southern Cross University, Lismore, NSW, Australia

**Keywords:** SIRT4, cellular metabolic, mitochondria, energy transfer, cell survival, exercise

## Abstract

NAD^+^-dependent SIRT4 has been reported to be a key regulator of metabolic enzymes and antioxidant defense mechanisms in mitochondria. It also plays an important role in regulation of mitochondrial metabolism in response to exercise. Recent studies have shown that SIRT4 is involved in a wide range of mitochondrial metabolic processes, including depressing insulin secretion in pancreatic beta cells, promoting lipid synthesis, regulating mitochondrial adenosine triphosphate (ATP) homeostasis, controlling apoptosis and regulating redox. SIRT4 also appears to have enzymatic functions involved in posttranslational modifications such as ADP-ribosylation, lysine deacetylation and lipoamidation. However, the effects on SIRT4 by metabolic diseases and changes in metabolic homeostasis such as during exercise, along with the roles of SIRT4 in the regulation of metabolism during disease, are not well understood. The main goal of this review is to critically analyse and summarise the current research evidence on the significance of the SIRT4 as a metabolic regulator and in mitochondrial function and its putative roles in relation to metabolic diseases and exercise.

## Introduction

Sirtuins (SIRTs) are highly conserved NAD (nicotinamide adenine dinucleotide)^+^-dependent deacetylases and ADP-ribosyl transferase with seven isoforms (SIRT1–7) that have been identified in mammals during the last 20 years (Verdin et al., 2010; Luo et al., 2017; Singh et al., 2017). The sirtuins are involved in many cellular processes, including metabolic homeostasis, antioxidant and redox signalling and ageing, and vary in subcellular localisation, enzymatic activity and targets (Houtkooper et al., 2012; Singh et al., 2017). SIRT3, 4 and 5 are mainly located in mitochondria and participate in many key metabolic processes of this organelle (Elkhwanky and Hakkola, 2018). Mitochondria play a key role in energy transfer, cell signalling and cell survival, while functional defects in mitochondria have been found to be associated with the ageing process and age-related diseases, such as metabolic diseases, cancer and neurodegeneration (Cobley et al., 2015; Osborne et al., 2016). These mitochondrial sirtuins have been shown to participate in the regulation of substance and energy metabolism, apoptosis and cell signalling (Haigis et al., 2006; Verdin et al., 2010).

SIRT3 has been a subject of intensive research as a mitochondrial fidelity protein, and its roles in mitochondrial substrate metabolism, protection against oxidative stress and cell survival pathways have been identified (Porter et al., 2014; Koentges et al., 2016; Osborne et al., 2016; Parodi-Rullán et al., 2017). SIRT5 has been shown to have the roles of desuccinylase, demalonylase and deglutarylase (Du et al., 2011; Peng et al., 2011; Tan et al., 2014) and appears to be an important regulator of the urea cycle (Elkhwanky and Hakkola, 2018). Although SIRT4 has not been widely studied as its homologues SIRT3, it is of great importance in basic mitochondrial biology (Zhu et al., 2014; Wood et al., 2018) and is considered as a nutrient sensing signal and negative regulator of mitochondrial metabolism (Zhu et al., 2014; Assadi-Porter et al., 2018; Tain et al., 2018; Wood et al., 2018). Due to its effects on ATP level and lipid metabolism, SIRT4 has been speculated to be associated with various mitochondrial dysfunction-related pathological conditions and diseases, including type 2 diabetes (T2D), nonalcoholic fatty liver disease, diet-induced obesity, apoptosis, inflammatory vascular disease and the development of various cancers (Saunders and Verdin, 2007; Laurent et al., 2013b; Wu et al., 2014; Osborne et al., 2016).

Exercise has been found to increase activities and protein levels of SIRT1 and SIRT3, decrease the level of lysine acetylation, increase the turnover rate of proteins, decrease the accumulation of carbonyl groups and improve cellular function (Radak et al., 2013b; Navas Enamorado, 2017). However, the effect of exercise on SIRT4 has not been investigated extensively, although it seems that SIRT4 is involved in the regulation of ATP production in exercise or physical activity (Marfe et al., 2010; Laurent et al., 2013b; Radak et al., 2013b; Parik et al., 2018).

Based on the background information above, this review is focused on a critical analysis of the known and potential roles of SIRT4 in regulation of cellular metabolic and energetic state, mitochondrial energy transfer, mitochondrial survival, redox regulation and metabolic diseases. In addition, the reports on the effects of exercise or physical activity on energy metabolism and mitochondrial homeostasis in relation to SIRT4 are also examined.

## SIRT4

The primary sequence and secondary structure of SIRT4 are quite similar to that of other mitochondrial sirtuins and contain the required sirtuin deacylase enzyme that catalyses deacylation reaction (Anderson et al., 2017). It has been proved to exist in the matrix of mitochondria in many organs including the heart, brain, kidney, liver and skeletal muscle (Haigis et al., 2006; Komlos et al., 2013; Osborne et al., 2016). Although SIRT4 has a highly conserved catalytic domain, it was initially reported to exhibit no NAD^+^-dependent deacetylase activity but to affect its targets mainly through NAD^+^-dependent ADP-ribosylation (Frye, 1999; Lombard et al., 2007). Recently however, SIRT4 has been reported to have specific deacetylase activity, which can deacetylate and inhibit malonyl CoA decarboxylase (MCD) (Laurent et al., 2013b). It is worth noting that SIRT4 is also considered as a cellular lipoamidase that inhibits pyruvate dehydrogenase (PDH) activity, and its catalytic efficiency for the modification of lidyl- and biotinyl-lysine is better than its deacetylation activity (Mathias et al., 2014). Furthermore, Anderson et al. (2017) have shown that SIRT4 also acts as a lysine deacylase that is involved in the control of leucine metabolism and insulin secretion.

In addition, SIRT4 is also involved in regulation of lipid metabolism and production of reactive oxygen species (ROS) in mitochondria (Singh et al., 2017; Elkhwanky and Hakkola, 2018). Fatty acid oxidation (FAO) can stimulate ROS production through upregulation of electron transport chain (ETC) activity. For example, FAO-driven H_2_O_2_ is a major source of oxidative stress and contributes to the development of certain diseases (Rosca et al., 2012; Zhang et al., 2019). SIRT4 has been explored as a biomarker for coronary artery disease (CAD) due to its association with increased mitochondrial ROS (Singh et al., 2017). Moreover, SIRT4 has a tumour-suppressive activity by inhibiting mitochondrial glutamine metabolism (Jeong et al., 2013). In short, since the expression of SIRT4 is different in different types of cells (Singh et al., 2017), it should be taken into account when detecting the changes of SIRT4 as a disease-specific biomarker and in response to exercise intervention.

## SIRT4 Regulation of the Cellular Metabolic and Energetic State Through NAD^+^

NAD^+^ and NADH play important roles in energy metabolism, cell death and various cell functions (Ying, 2006). Mitochondria contain a major portion of intracellular NAD^+^ (Tischler et al., 1977). NAD^+^ is an important redox cofactor and can act as a cosubstrate for a series of enzymes. Within the mitochondria, NAD^+^ accepts electrons and transfers them to ETC for production of ATP (Davila et al., 2018). The levels of NAD^+^ and NADH have a regulatory effect on mitochondrial sirtuin activity (Verdin et al., 2010). Previous studies have suggested that mitochondria regulate NAD^+^ and NADH levels through mitochondrial permeability transition pore (mPTP), that is, opening mPTP will lead to the release of mitochondrial NAD^+^ from matrix space to intermembrane space which is subsequently hydrolysed by NAD^+^ glycosylase (Di Lisa et al., 2001). However, more recent research presents evidence that NAD^+^ can be directly imported into mitochondria from the cytoplasm, challenging the conventional view that the mitochondrial inner membrane is impermeable to pyridine nucleotides, and suggesting the presence of a new NAD^+^ (or NADH) transporter (Davila et al., 2018). NAD^+^ produces multiple biological effects through a variety of NAD^+^-dependent enzymes, including sirtuins (Ying, 2006). However, NADH is not a common substrate for sirtuins; and a change of NAD^+^/NADH ratio can alter the redox state of cells, alter the activity of polymerase (PARPs) and sirtuins, and further affect the signalling cascades and gene expression (Ying, 2006; Ho et al., 2013). NAD^+^-dependent protein deacetylase and ADP-ribosyltransferase are two major forms of sirtuins (Verdin et al., 2010). SIRT4 contains the requisite amino acids to participate in the deacylase reaction and homologous sirtuin deacylase domain, a conserved catalytic histidine (H161) and a Rossmann fold NAD^+^-binding motif (Min et al., 2001; Anderson et al., 2017). In a state of nutritional deficiency, sirtuin activity increases when NAD^+^ levels are high (Yang et al., 2007). This view is supported by mass spectrometry studies that have revealed that metabolic proteins, such as TCA cycle enzymes, fatty acid oxidation enzymes and subunits of oxidative phosphorylation (OXPHOS) complexes, are acetylated in response to metabolic stress (Schwer et al., 2009; Zhao et al., 2010). There has been evidence that an increased level of mitochondrial NAD^+^ has a protective effect in cell survival under genotoxic stress through mitochondrial sirtuins SIRT3 and SIRT4 (Yang et al., 2007). Unlike other mitochondrial sirtuins, SIRT4 catalyses the transfer of ADP-ribose from NAD^+^ to target proteins. For example, SIRT4 can downregulate insulin secretion from pancreatic beta cells by using NAD to ADP-ribosylate and depress the glutamate dehydrogenase activity (Haigis et al., 2006). In contrast to the functions of SIRT1 and SIRT3 as promoters of oxidative capacity, SIRT4 is reported as a negative regulator of oxidative metabolism (Nasrin et al., 2010). In the study by Nasrin et al. (2010), although it was unclear how SIRT4 was dependent on SIRT1 to regulate fatty acid metabolism, the authors speculated that it could be related to the NAD^+^ conversion efficiency, ATP consumption and even generation of peroxisome proliferator-activated receptor α (PPARα) ligands.

Physical exercise can effectively improve the mitochondrial function of healthy elderly people (Cobley et al., 2015; Park et al., 2016), which is partly related to the changes in NAD^+^/NADH ratio caused by different energy demands (Hipkiss, 2010). By changing the NAD^+^/NADH ratio to improve mitochondrial function, physical exercise may alter the activity of some NAD^+^-dependent sirtuins (Navas Enamorado, 2017). Radak et al. (2013b) proposed that exercise could significantly change the expression, content and activity of SIRT1 and SIRT3. Based on this information, we hypothesise that physical exercise also has a beneficial effect on SIRT4 activity by increasing the NAD^+^/NADH ratio.

## SIRT4 Regulation of Mitochondrial Energy Transfer and Cell Survival

### Mitochondrial Adenosine Triphosphate Homeostasis

Maintenance of energy balance is a vital aspect of homeostatsis. Energy balance in cells is regulated by multiple mechanisms, and mitochondria are the main powerhouse of the cell. The roles of mitochondrial sirtuins (SIRT3–5) have been extensively discussed elsewhere (Huang et al., 2010; Osborne et al., 2016; Tang et al., 2017; Elkhwanky and Hakkola, 2018; Parodi-Rullán et al., 2018). SIRT4 has been shown to regulate mitochondrial ATP homeostasis by affecting mitochondrial uncoupling with ANT2 (adenine nucleotide translocation agent 2) (Ho et al., 2013). The ANT is an ADP/ATP translocation agent, which is responsible for the transport of ATP from mitochondrial matrix to cytoplasm and ADP from cytoplasm to the matrix, and can also serve as the substrate of SIRT4 (Ahuja et al., 2007). Acylated ANT uncouples mitochondria (in this state, energy substrates such as fatty acids are oxidised and oxygen is consumed without ATP synthesis) and reduces the efficiency of OXPHOS, and the uncoupling in the liver predominantly depends on ANT2 (Klingenberg, 2008). It has been shown that SIRT4 regulates ANT2-mediated OXPHOS efficiency, with the overexpression of SIRT4 is related to the increase of ATP level and the absence of SIRT4 is associated with mitochondrial uncoupling, thus increasing oxygen consumption (Ho et al., 2013). The underlying mechanism may be that SIRT4 deficiency could initiates retrograde signalling response from mitochondria to the nucleus to regulate ATP levels through ANT2/AMPK/PGC-1α (peroxisome proliferator-activated receptor-γcoactivator-1α) signalling pathway (Ho et al., 2013). However, it seems that SIRT4 and SIRT3 regulate mitochondrial ATP homeostasis through different mechanisms. SIRT3 increases ATP production by deacetylating ETC enzymes thus increasing respiration (Ahn et al., 2008; Laurent et al., 2013a), while SIRT4 requires ANT to affect cellular ATP level (Parihar et al., 2015). Therefore, to examine the role of NAD^+^-dependent SIRT4 in mitochondria may provide instructive clues to clarify the regulatory mechanism of mitochondrial ATP homeostasis.

Skeletal muscle contractile capacity is closely related to ATP production, NAD^+^ homeostasis and pyrimidine nucleotide synthesis in mitochondria (Navas Enamorado, 2017). A plethora of studies suggests that an increase of ADP/ATP ratio in skeletal muscle during physical activity induces AMPK activity, thereby regulates the β-oxidation capacity and mitochondrial biogenesis (Winder et al., 1997; Zong et al., 2002). Together, based on these findings, we speculate that SIRT4 plays an important role in mitochondrial ATP homeostasis during exercise.

### Fatty Acid Oxidation

**Figure 1:**
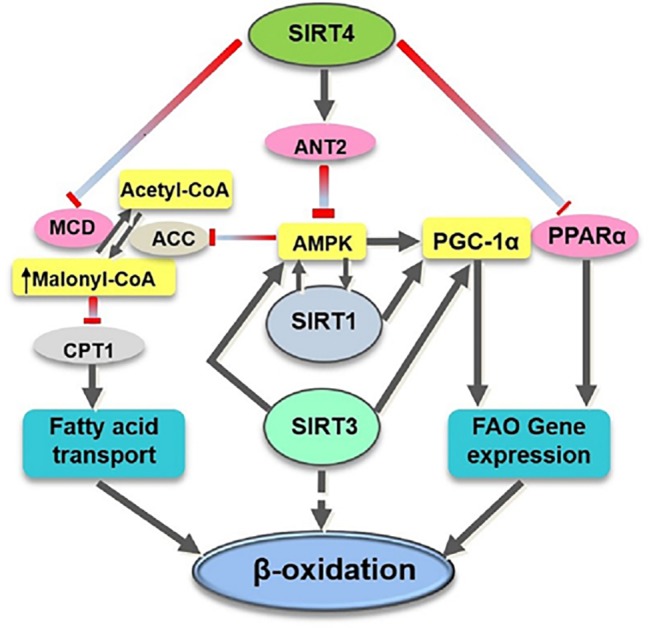
Overview of the target substrates of SIRT4, SIRT1, and SIRT3 involved in fatty acid oxidation. SIRT4 directly (pink ovals) or indirectly (yellow rectangles) modulates the activity of various target substrates involved in fatty acid metabolism. ACC, acetyl CoA carboxylase; CPT1, carnitine palmitoyltransferase 1; FAO, fatty acid oxidation.

SIRT4 is found to be one of the critical regulators for lipids metabolism, regarded as the mitochondrial metabolic break and the gatekeeper of the lipids metabolism (Elkhwanky and Hakkola, 2018). It has been demonstrated that SIRT4 has deacetylase activity to deacetylate and inactivate malonyl CoA decarboxylase (MCD) which promotes the production of acetyl CoA from malonyl CoA (Laurent et al., 2013b). It has been reported that, in SIRT4 knocked-out (KO) mice, the MCD activity was increased and the level of malonyl CoA was decreased in skeletal muscle and white adipose tissue, leading to elevated exercise tolerance, which was considered to have a protective effect on diet-related obesity (Laurent et al., 2013b). Another mechanism for SIRT4 to regulate fatty acid metabolism may be at least partially dependent on SIRT1 because SIRT1 is essential for the enhanced oxidative capacity in SIRT4-deficient cells (Nasrin et al., 2010). Moreover, Nasrin et al. (2010) suggested that SIRT4 affects AMPK-SIRT1 pathway and contributes to the energy metabolism (Figure 1).

SIRT4 has also been shown to suppress hepatic fat oxidation by decreasing PPARα activity and consequently expression of PPARα target genes in a cell-autonomous manner (Laurent et al., 2013a). PPARα is a ligand-activated transcription factor that has been shown to promote the transcription of genes involved in fatty acid catabolism (Leone et al., 1999). Mice lacking PPARα failed to maintain metabolic homeostasis during fasting and displayed reduced levels of fatty acid oxidation (Kersten et al., 1999). The crosstalk between mitochondrial and nuclear sirtuins may be important to regulate fatty acid oxidation because deletion of SIRT4 activates PPARα activity through activation of SIRT1 by NAD^+^ (Figure 1; Laurent et al., 2013a).

In contrast to SIRT3’s positive role in improving fatty acid oxidation (Hirschey et al., 2010), SIRT4 acts as a negative regulator of fatty acid oxidation (Parihar et al., 2015). A decreased level of SIRT3 is reported to be involved in impaired FAO in skeletal muscle and reduced exercise capacity in heart failure (Tsuda et al., 2018). The SIRT3 and SIRT4 appear to have opposing roles in the regulation of fatty acid oxidation (Hirschey et al., 2010; Nasrin et al., 2010) that requires further study to determine how these two NAD^+^-dependent mitochondrial enzymes modulate different metabolic responses in similar states (Figure 1).

Regular, moderate level of exercise has been shown to increase skeletal muscle NADH and fatty acid oxidation levels in overweight older adults (Menshikova et al., 2017). Exercise improves mitochondrial capacity to oxidise fatty acids in skeletal muscle, which is beneficial to the health of obese individuals (Holloway, 2009). Furthermore, exercise has been observed to increase SIRT3 expression (Palacios et al., 2009). Another study found that exercise training can regulate the expression of SIRT3 but not SIRT1 in skeletal muscle (Palacios et al., 2009). Although both SIRT3 and SIRT1 can promote mitochondrial biogenesis and fatty acid oxidation through PGC-1α, they act in different pathways: SIRT3 promotes the gene expression of PGC-1α while SIRT1 directly activates PGC-1α by deacetylation (Gerhart-Hines et al., 2007; Palacios et al., 2009). It has been reported that the lipid metabolism of SIRT4 KO mice was deregulated, leading to a metabolic shift toward lipid utilisation, thus enhancing exercise tolerance and protecting against diet-induced obesity (Laurent et al., 2013b). However, little is known about the effects of exercise on SIRT4 expression and the involved factors and pathways in skeletal muscle.

### Apoptosis

Apoptosis is a process of programmed cell death. Accelerated apoptosis has been shown to occur in various disease status, but inhibition of apoptosis may lead to cancer and certain viral infections (Phaneuf and Leeuwenburgh, 2001). Permeability activation of mitochondrial outer membrane is an important event in cell apoptosis, which represents the terminal point of irreversible cell death and induces the release of caspase-activated molecules, the production of caspase-independent death effect factors and the collapse of ATP production (Verdin et al., 2010).

Although mitochondria play a central role in controlling apoptosis, few studies have been conducted on how mitochondrial sirtuins are involved in apoptosis (Verdin et al., 2010). It has been reported that SIRT3 and SIRT4 display anti-apoptotic activity with Nampt, an NAD^+^ biosynthetic enzyme, to protect cells from death under the condition of nutrient restriction (Yang et al., 2007). In addition, SIRT4 is a key player involved in hypoxia-induced apoptosis of cardiac myocytes, which can affect the ratio of pro-caspase-9/caspase-9 or pro-caspase-3/caspase-3, regulate Bax translocation, and thereby change the development and viability of H9c2 cell apoptosis (Liu et al., 2013). SIRT3 and SIRT4 have been shown to induct cardio protection of rhamnetin, protecting cells against H_2_O_2_-induced apoptosis in H9c2 cardiomyoblasts without any cytotoxicity (Park et al., 2014).

**Figure 2:**
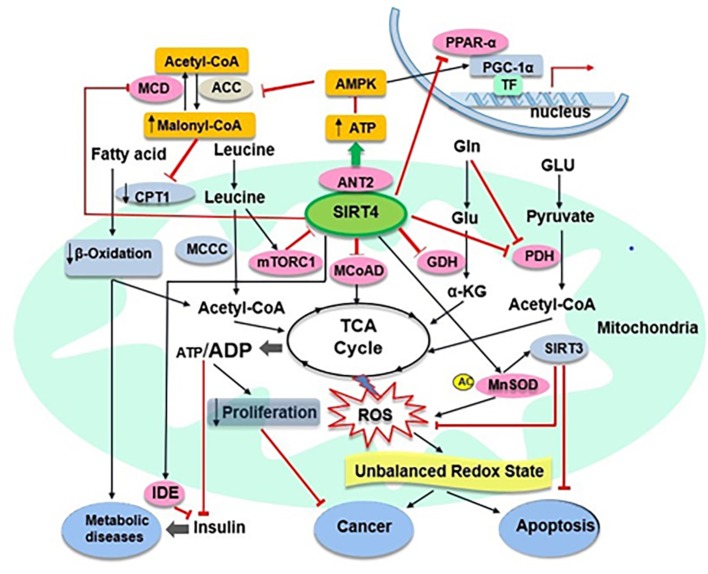
SIRT4 and its target substrates in mitochondrial metabolic pathways. SIRT4 directly (pink ovals) or indirectly (abricot rectangles) modulates the activity of various target substrates, that play crucial roles in mitochondrial metabolic processes. Black arrows and red lines indicate the promotion or suppression of a particular activity, respectively. α-KG, α-ketoglutarate; MCoAD, malonyl CoA decarboxylase; GLU, glucose; Gln, glycogen; TF, transcription factors.

Interestingly, it becomes clear that SIRT3 and SIRT4 play the same role in controlling apoptosis but they have opposite effects in regulating fatty acid metabolism, as discussed above (Nasrin et al., 2010). A recent study has shown that overexpression of SIRT4 may inhibit podocyte apoptosis and reduce podocyte injury *via* increasing the mitochondrial membrane potential (MMP) and decreasing ROS from mitochondrial sources in podocytes under hyperglycaemic conditions (Shi et al., 2017). SIRT4 overexpression also may downregulate the expression of apoptosis related proteins NOX1, Bax and phosphorylated p38 and upregulate the expression of Bcl2 in glucose simulated podocytes (Shi et al., 2017). Therefore, the intervention and regulation of SIRT4 may provide a new targeted strategy for the treatment of multiple diseases caused by apoptosis. Recently, several studies have shown that SIRT4 acts as a tumour suppressor *in vitro* and *in vivo* by regulating glutamine metabolism (Csibi et al., 2013; Jeong et al., 2013, 2014). However, in a conflicting report, SIRT4 showed oncogenic properties, which protected cancer cells against stress-induced apoptotic cell death, such as induced by DNA damage and endoplasmic reticulum stress. In that study, cells with SIRT4 overexpression appeared to have reduced cell death, correlating with reduced levels of leaved caspase-3, a marker of apoptosis (Jeong et al., 2016). Together, these results suggest that SIRT4 may play an important role in controlling cell apoptosis and may have the protective function against cell death even in cancer cells (Figure 2). Further studies are needed to determine the role of SIRT4 in the balance between anti-stress (anti-apoptosis) and tumour inhibition (pro-apoptosis) under different physiological conditions and the exact mechanism involved.

During or after an exercise, some factors may activate apoptosis in the body, but the exact mechanism of apoptosis is still unclear, which may be related to different cell types or stress stimuli. It has been suggested that exercise-induced apoptosis is a normal regulatory process that clears certain damaged cells without significant inflammatory response, thus ensuring optimal body function (Phaneuf and Leeuwenburgh, 2001). However, whether SIRT4 is involved in exercise-induced apoptosis has not been reported, which will be an interesting question worth studying in the future.

### Insulin Secretion

SIRT4 has been shown to repress insulin secretion in response to glucose and amino acids stimulation (Argmann and Auwerx, 2006; Ahuja et al., 2007). There has been a report that SIRT4 can modify GDH to affect insulin secretion and amino acid metabolism as a mitochondrial ADP-ribosyltransferase in pancreas, liver and brain (Haigis et al., 2006). GDH, a mitochondrial enzyme, is known to convert glutamate into the TCA-cycle intermediate α-ketoglutarate, generating ATP (Herrero-Yraola et al., 2001). The increase of ATP level leads to the closure of ATP-sensitive K^+^ channel, which in turn depolarises the cell membrane, opens voltage-gated L-type Ca^2+^ channels and finally promotes insulin secretion (Argmann and Auwerx, 2006). GDH is the first discovered substrate of SIRT4, and its activity is inhibited by ADP-ribosylation (Parihar et al., 2015). In wild-type mice, SIRT4 ADP-ribosylates and downregulates the enzymatic activity of GDH, thereby preventing insulin secretion in pancreatic β cells in response to glutamine and leucine, whereas glutamine induces insulin secretion in SIRT4 knockout mice (Haigis et al., 2006). Furthermore, SIRT4 promotes leucine catabolism through upregulation of methylcrotonyl-CoA carboxylase (MCCC) and inhibits glutaminolysis *via* regulation of GDH (Anderson et al., 2017). Active SIRT4 reduces mitochondrial fuel oxidation into the TCA cycle, leading to the decrease of ATP/ADP ratio and eventually inhibiting insulin secretion (Zaganjor et al., 2017). Insulin-degrading enzyme (IDE) and ADP/ATP carrier protein ANT2 have been proved as two other substrates of SIRT4, that are involved in the secretion of insulin in response to glucose (Ho et al., 2013; Osborne et al., 2016). IDE is a zinc metalloprotease widely expressed to regulate cerebral amyloid β peptide levels and plasma insulin levels (Farris et al., 2003; Miller et al., 2003). SIRT4 is highly expressed in β cells of the islet, interacting with the ANT2/3 and IDE protease, and negatively regulates insulin secretion in response to glucose (Ahuja et al., 2007). It is further reported that SIRT4 is detected without any deacetylase activity but has a strong and reproducible ADP-ribosyltransferase activity, further supports the role of SIRT4 in inhibiting insulin secretion in pancreatic β cells (Ahuja et al., 2007).

SIRT4 appears to have lipoamidase activity and inhibits PDH as another molecular target that also negatively regulates acetyl-CoA production (Mathias et al., 2014). PDH is known to convert pyruvate to acetyl-CoA, being primarily regulated by phosphorylation of its E1 component. SIRT4 may enzymatically hydrolyse the lipoamide cofactors from the E2 component dihydrolipoyllysine acetyltransferase (DLAT) and then diminish PDH activity (Mathias et al., 2014). This will help to further understand the insulin secretion initiated by the rapid utilisation of glucose for glycolysis and the consequent entry into the TCA cycle for OXPHOS (Newgard and McGarry, 1995). A recent study showed that SIRT4, as a protein deacylase, could act on lysine modifications derived from reactive acyl species of leucine catabolism (Anderson et al., 2017). A comprehensive analysis of phylogenetics, structural biology and enzymology showed that SIRT4 removes three acyl moieties from lysine residues, including methylglutaryl (MG)-, hydroxymethylglutaryl (HMG)-, and 3-methylglutaconyl (MGc)-lysine (Zaganjor et al., 2017). Mice lacking of SIRT4 had abnormal leucine metabolism, which resulted in chronic increase of insulin secretion and accelerated senescence-induced insulin resistance (Anderson et al., 2017). These findings indicate some robust enzymatic activities for SIRT4 in relation to the mechanisms in regulation of insulin secretion, and the role of SIRT4 as a crucial player maintaining insulin secretion and glucose homeostasis and cellular metabolism (Figure 2). Future studies are needed to elucidate how SIRT4 regulates insulin secretion by harmonising these enzymes.

It is well known that increasing physical activity and changing sedentary lifestyle can effectively reduce and/or delay the development of IR (Insulin resistance), providing evidence for the application of exercise in the prevention and treatment of type 2 diabetes (Fedewa et al., 2014). Laurent et al. (2013b) have reported that SIRT4 KO mice showed increased exercise tolerance, suggesting that SIRT4 may be involved in metabolic reprogramming during exercise training. Because high mitochondrial flux and increased fatty acid oxidation are associated with the phenotypes of insulin resistance model (Koves et al., 2008; Sunny et al., 2011); therefore, it will be of great significance to clarify the role of SIRT4 in regulating glucose and insulin homeostasis and to further investigate the potential therapeutic effects of modulating SIRT4 function by exercise.

### Mitochondrial Oxidative Stress and Antioxidant Response

Mitochondria play a key role in cell survival and death (Parihar et al., 2015). Disorder of mitochondrial energy metabolism underlies cellular oxidative stress and ROS production (Beal, 2005). ROS are natural by-products of oxygen metabolism, mainly derived from oxidative phosphorylation in mitochondria, enzymatic reactions, unfolded protein reactions and peroxisomes (Schiattarella and Hill, 2017). Mitochondrial complex I and complex III are the major ROS sources under pathological conditions (Aon et al., 2003), while NADPH-oxidase, monoaminoxidase and alpha-glycerophosphate dehydrogenase and other enzymes are also mitochondrial ROS production sites under physiological conditions (Zorov et al., 2014). Moderate levels of ROS play important roles in physiology, such as signal transduction and stress responses (Schiattarella and Hill, 2017). However, both high and excessively low levels of ROS are harmful and play a significant pathogenic role in the process of dysfunction caused by drastic changes in the oxidative environment (Stanley et al., 2011; Aon et al., 2012; Zorov et al., 2014). SIRT4 has been shown to be involved in the regulation of mitochondrial ROS production in Ang II-induced pathological cardiomyocytes (Luo et al., 2017). The levels of ROS in both heart and mitochondria decreased in SIRT4-KO mice and increased in SIRT4 overexpression mice, respectively. These results were also observed in rat cardiomyocytes, suggesting that SIRT4 may directly control the production of ROS to some extent in heart muscle cells (Luo et al., 2017). Luo et al. (2017) and Schiattarella and Hill (2017) reported that SIRT4 competed with SIRT3 for manganese superoxide dismutase (MnSOD) binding; MnSOD-bound SIRT4 did not promote MnSOD deacetylation, and the hyperacetylated enzyme was relatively inactive, thus leading to ROS accumulation, hence promoting pathological cardiac hypertrophy remodelling. SIRT3 has been shown to deacetylate MnSOD or ICDH2 (isocitrate dehydrogenase 2), which induces increased antioxidant activities of these enzymes and decreased cell ROS levels (Parihar et al., 2015). However, another study found that overexpression of SIRT4 prevented glucose-induced podocyte apoptosis *via* the mitochondrial pathway, accompanied by increased mitochondrial membrane potential and reduced ROS production (Shi et al., 2017). Interestingly, these findings reveal that SIRT4 may induce ROS production but has an antioxidative role as well (Singh et al., 2017).

Also, circulating levels of SIRT4 has been explored as a potential biomarker of oxidative metabolism for CAD in patients with obesity (Tarantino et al., 2014). Compared with healthy subjects, obese patients with hepatic steatosis and increased intramuscular triglyceride (IMTG) had significantly lower levels of circulating SIRT4, which was strictly related to some parameters reckoned as CAD risk factors. Low circulating levels of SIRT4 in obese patients suggest that there was a reduced SIRT4 expression in mitochondria that would increase the fat oxidation capacity and the mitochondrial function in liver and skeletal muscle (Tarantino et al., 2014). Because fatty acid oxidation is closely related to the production of mitochondrial ROS (Rosca et al., 2012; Cortassa et al., 2017), SIRT4 modulation of fatty acid metabolism can reduce high circulating levels of free fatty acids but unfortunately increase ROS production in obese patients with nonalcoholic fatty liver disease (Tarantino et al., 2014). Another study on SIRT4 levels in obese individuals adhering to the Mediterranean diet found that average adherers had higher SIRT4 circulating levels than low adherers, accompanied with lower ectopic fat storage and adipocyte dysfunction independent to body mass index (BMI), which agrees with the fact that the circulating levels of SIRT4 are negatively correlated with energy intake and positively correlated with the intake of antioxidant vitamins and minerals (Barrea et al., 2017).

It is noteworthy that a recent study revealed a new novel function of SIRT4 in mitochondrial quality control and mitophagy regulation through interaction with OPA1 (optic atrophy 1) (Lang et al., 2017). In this study, moderate overexpression of SIRT4 not only promoted stress-induced (CCCP-triggered dissipation of the mitochondrial membrane potential) mtROS production in HEK293 cells *via* its enzymatic activity but also decreased Parkin expression associated mitophagy. Moreover, upregulation of endogenous SIRT4 expression in fibroblast models of senescence increased L-OPA1 levels and mitochondrial fusion in a SIRT4-dependent manner, suggesting that SIRT4 interacted with L-OPA1 as a novel determinant to down-regulate mitophagy in ageing process by shifting the mitochondrial fusion/fission cycle towards fusion (Lang et al., 2017). Together, these results suggest that SIRT4 may play an important role in managing the factors involved in the stress and antioxidant response (Figure 2). However, regarding the expression of SIRT4 in different types of cell, further studies are needed to determine the full capabilities and actions of SIRT4 in antioxidant responses and oxidative stress, as well as in oxidative stress-associated disease conditions (Singh et al., 2017).

The relationship between exercise and oxidative stress is very complicated due to the different mode, intensity and duration of exercise (Pingitore et al., 2015). Regular training at moderate level seems to be beneficial for reducing oxidative stress and promoting health; however, vigorous aerobic and anaerobic exercises lead to increased oxidative stress, although they can also improve the endogenous antioxidant defense system (Pingitore et al., 2015). It remains to be examined whether SIRT4 can serve as a regulator of antioxidant response during exercise.

### Metabolic Diseases and Cancer

It is known that SIRT4 has effects on substrates metabolism, therefore been speculated to be associated with pathological conditions and diseases (Mathias et al., 2014; Osborne et al., 2016). Repressing the GDH activity by SIRT4 has been suggested to protect against the development of T2D (Saunders and Verdin, 2007). Furthermore, it has been found that SIRT4 mRNA levels in granulocytes and monocytes were significantly lower in T2D group than that in the normal control group (Song et al., 2011). The findings for the T2D group showed that SIRT4 mRNA expression in peripheral blood leukocytes was negatively correlated with high-density lipoprotein cholesterol (HDL) level and positively correlated with triglyceride/lipoprotein a level (Song et al., 2011); absence of SIRT4 is likely to enhance the development of T2D (Mahlknecht and Voelter-Mahlknecht, 2009). On one hand, serum SIRT4 in obese patients was low and showed negative correlations with anthropometric and metabolic parameters (such as BMI, waist circumference, cardiovascular risk factors) but significant positive correlation with peak GH (growth hormone) and IGF-1 (insulin-like growth factor), likely representing a compensatory mechanism to deal with the negative regulator role played by SIRT4 in mitochondrial oxidative capacity (Savastano et al., 2015). On the other hand, increased SIRT4 activity is likely to inhibit fatty acid oxidation and promote ectopic lipid storage (Nasrin et al., 2010). SIRT4 expression was upregulated in the liver of rats with high-fat diet, while no significant changes in the caloric restriction (CR) diet group compared with the control group (Chen et al., 2010). Meanwhile, a study found that nonalcoholic fatty liver disease patients exhibited increased SIRT4 expression and potentially a decrease in fatty acid oxidation in the liver (Wu et al., 2014). These findings suggest that SIRT4 is related to the occurrence and development of metabolic diseases such as insulin resistance, T2D, obesity and nonalcoholic fatty liver disease (Figure 2). Therefore, further research is needed to identify the possible beneficial roles of SIRT4 modulation in relation to metabolic diseases.

In addition to the regulation of mitochondrial metabolic functions, deregulated metabolism is tightly connected with the development of various cancers (Osborne et al., 2016). SIRT4 seems to have tumour suppressive activity induced by multiple genotoxic agents, and SIRT4-dependent inhibition of mitochondrial glutamine metabolism is necessary for the proper implementation of cell DNA damage response program (Gertz and Steegborn, 2016). Further, it has been reported that SIRT4 expression was reduced in patients with cancers such as colon and gastric cancers (Csibi et al., 2013).

SIRT4 is also involved in the development of brain astrocytes. SIRT4-dependent inhibition of GDH is believed to regulate the development of glial cells (Komlos et al., 2013). A recent study evaluated the role of SIRT4 with oncogenic or tumour-suppressive activity in cancer, suggesting that inhibiting metabolism and regulation of genome stability DNA were two possible mechanisms for the role of SIRT4 in tumours (Huang and Zhu, 2018). As mentioned above, SIRT4 may also promote Ang II-induced pathological cardiac hypertrophy by inhibiting the activity of manganese superoxide dismutase (Schiattarella and Hill, 2017). Together, these results suggest that SIRT4 is a potential regulator of the activation of certain compounds in cancer therapy and to play an important role in managing the players involved in the antioxidant responses.

## SIRT4 and Exercise

Accumulated experimental data have shown that exercise can increase oxygen consumption and rebuild the balance of intracellular pro-oxidant-antioxidant homeostasis (Radak et al., 2013b). The mitochondrial ETC and xanthine oxidase have been identified as the major sources of intracellular ROS (Amélie et al., 2011). Sirtuins have been recognised as redox-sensitive energy sensors during exercise, especially for SIRT1, an exercise-associated oxidative challenge can result in its higher expression, content, and activity in humans (Radak et al., 2011; Conti et al., 2012; Carrico et al., 2018). For mitochondrial sirtuins, SIRT3 is readily modified by physical exercise. For example, exercise training can result in an increase in SIRT3 protein content in both murine and human skeletal muscle (Brandauer et al., 2015; Vargas-Ortiz et al., 2015). In contrast to other sirtuins, understanding of the functions and targets of SIRT4 in exercise is still very limited. Due to its complex effects on mitochondrial energy metabolism and mitochondrial adaptive capacity to exercise, it is very likely that SIRT4 can be modulated by exercise.

Two studies have reported that a 12-week treadmill training significantly decreased SIRT4 level in gastrocnemius muscle of rats that were selectively bred as low-capacity runners (LCRs) and high-capacity runners (HCRs), respectively, suggesting decreased SIRT4 level resulted in increased free fatty acid utilisation that is a key element for elevating endurance capacity (Hart et al., 2013a,b). These results also indicate that the downregulating effects of exercise training on SIRT4 content could have beneficial effects on glucose handling because SIRT4 is shown to be involved in the development of insulin resistance (Chen et al., 2010). Recently, it has been reported that SIRT4 KO mice can run 20% more distance and longer time than wild-type mice facing a graded, maximal treadmill challenge, which may be related to increased fatty acid oxidation, enhanced exercise capacity and resistance to diet-induced obesity (Laurent et al., 2013b). Since the decrease of malonyl CoA can increase fat oxidation in muscles after exercise (Dean et al., 2000), SIRT4 knockout leads to the decrease of malonyl coenzyme levels and the increase of exercise capacity, indicating that SIRT4 may play a role in the metabolic reprogramming during exercise training (Laurent et al., 2013b).

Karvinen et al. (2016) found that the level of SITR4 protein was unchanged in rat skeletal muscle after a 1-year voluntary aerobic wheel running. However, a study of mountaineers showed that the SIRT4 mRNA level in the skeletal muscle elevated after 5 weeks exposure to high altitude which included climbing peaks over 8,000 m, suggesting that SIRT4 is involved in the changes in fatty acid metabolism at high altitude and physical activity (Radak et al., 2013a; Acs et al., 2014). A study on *Drosophila melanogaster* has found that loss of mitochondrial SIRT4 shortened the insects’ lifespan and decreased their physical activity (Parik et al., 2018).

**Figure 3:**
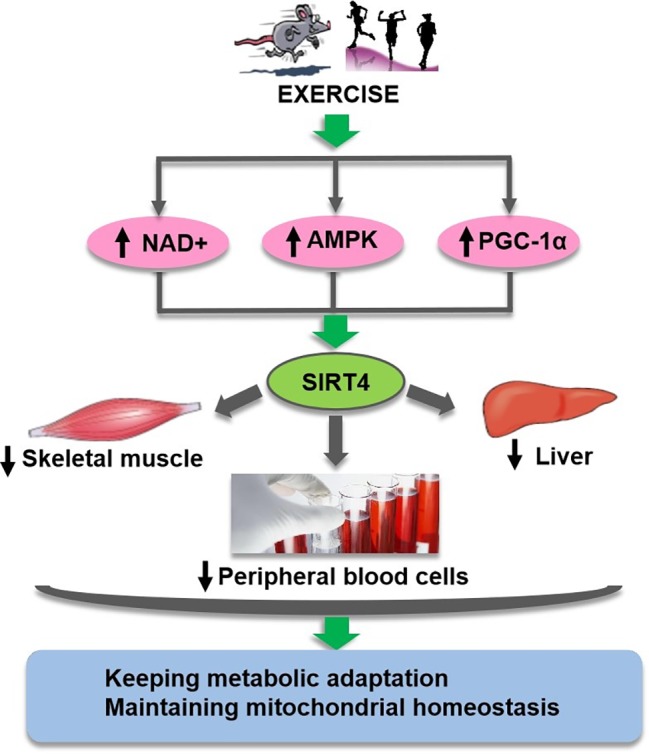
Exercise regulates SIRT4 through suggested multiple pathways.Exercise may regulate SIRT4 (green oval) activity in different tissues and cells through complex pathways (pink ovals) mediated by NAD+, AMPK, and PGC-1ɑ, so as to keep metabolic adaptation and maintain mitochondrial homeostasis.

In summary, these reports suggest that NAD^+^-dependent SIRT4 is very sensitive to metabolic challenges and may be modulated by physical exercise, although there have been debates about the expression of SIRT4 depending on the cell type (Figure 3). It is of a compelling interest to elucidate the regulatory role of SIRT4 on mitochondrial metabolism and its relationship with physical fitness, health and ageing.

## Concluding Remarks

Research on animal models at the cellular level together with the data obtained from human studies suggest that SIRT4 is a master regulator of a wide range of mitochondrial metabolic processes in mammalian cells, such as depressing insulin secretion, promoting lipid synthesis, regulating mitochondrial ATP homeostasis, controlling apoptosis and regulating redox. SIRT4 could be on both sides of redox regulation and is the only sirtuin shown to induce ROS production. Exercise can activate a number of pathways that contribute to metabolic reprogramming and result in an increased generation of ROS that can modulate muscle contraction, antioxidant protection, and oxidative damage repair. The adaptations induced by exercise are typically expected to protect against metabolic and age-related diseases and improve health, making it important to identify proteins involved in this reprogramming for treatment of metabolic diseases. Although the effects of exercise on SIRT4 have not been thoroughly investigated, it appears that SIRT4 might play a role in metabolic regulation and control during exercise. Further research is thus required to elucidate the roles of SIRT4 in relation to the benefits of exercise in prevention and treatment of metabolic diseases.

## Author Contributions

YH and SZ were the primary authors who were responsible for the design and writing of the manuscript. SC and AC assisted with writing and editing the manuscript.

### Conflict of Interest Statement

The authors declare that the research was conducted in the absence of any commercial or financial relationships that could be construed as a potential conflict of interest.
